# Categorisation of the One Welfare Practices in Beekeeping

**DOI:** 10.3390/ani15152236

**Published:** 2025-07-30

**Authors:** Claudia Mortellaro, Elena Giannottu, Camilla Pedrelli, Valentina Lorenzi, Marco Pietropaoli, Veronica Manara, Martina Girola, Alessandra De Carolis, Marina Bagni, Giovanni Formato

**Affiliations:** 1 Istituto Zooprofilattico Sperimentale del Lazio e Della Toscana “M. Aleandri”, International Cooperation and Research for Sustainable Development in Beekeeping Laboratory, FAO Reference Centre, “Animal Health and Food Security Discipline Apiculture, Health and Biosecurity”, WOAH Collaborating Centre, “Good Beekeeping Management Practices and Biosecurity Measures in the Apiculture Sector”, Via Appia Nuova 1411, 00178 Rome, Italy; camilla.pedrelli-esterno@izslt.it (C.P.); marco.pietropaoli@izslt.it (M.P.); veronica.manara-esterno@izslt.it (V.M.); martina.girola-esterno@izslt.it (M.G.); alessandra.decarolis-esterno@izslt.it (A.D.C.); giovanni.formato@izslt.it (G.F.); 2 Indipendent Veterinary Practitioner, 00147 Rome, Italy; giannottue@gmail.com; 3 Istituto Zooprofilattico Sperimentale della Lombardia e dell’Emilia-Romagna “Bruno Ubertini”, Italian Reference Centre for Animal Welfare (CReNBA), Via Bianchi 9, 25124 Brescia, Italy; valentina.lorenzi@izsler.it; 4 Ministry of Health, Viale Giorgio Ribotta 5, 00144 Rome, Italy; m.bagni@sanita.it

**Keywords:** one welfare, one welfare practices, beekeeping, honey bee welfare, human wellbeing, environmental welfare

## Abstract

Honey bees are vital to ecosystem conservation, agricultural production, and biodiversity, yet their welfare has often been overlooked. This study introduces the integration of Honey Bee Welfare Practices (HBWPs) into the One Welfare framework, addressing the interconnectedness of honey bee welfare, environmental welfare, and human wellbeing. We analysed and re-evaluated the 243 HBWPs already identified and categorised within the context of the Five Domains model in 2024 by Giovanni Formato et al., and we explored their broader impacts. By incorporating the One Welfare approach, we assessed each practice’s effect on bee welfare both as individuals and as a superorganism, human wellbeing, and environmental welfare, as well as their economic and time-related implications for beekeepers. The aim of this study was to obtain a list of One Welfare Practices in Beekeeping, considering all stakeholders as equally important. The analysis highlights the multidimensional nature of beekeeping, with 280 practices positively affecting honey bee welfare, while also considering their potential impact on human wellbeing, environmental health, and production. Challenges such as balancing beekeeper time constraints and welfare goals are discussed, with recommendations for practical compromises. This approach can offer a holistic and sustainable model for apiculture, ensuring that welfare is maintained across all stakeholders, and provides a flexible framework applicable to various beekeeping systems worldwide.

## Simple Summary

Honey bees are essential for agriculture and biodiversity. However, their welfare is often overlooked. This study explores how Honey Bee Welfare Practices (HBWPs) can be integrated into the One Welfare framework, which considers animal, human, and environmental welfare as equally important. We re-evaluated 243 previously identified HBWPs and analysed their impact not only on honey bee welfare, but also on human wellbeing and environmental sustainability. We also considered how each practice affects productivity, time demands for beekeepers, and the attitude of beekeepers toward animal welfare. As a result, we defined a new set of “One Welfare Practices in Beekeeping”. These practices support a balanced and inclusive approach to beekeeping, where the needs of bees, people, and the environment are all taken into account. While some practices require time and effort, they can lead to long-term benefits such as healthier colonies, improved working conditions, and environmental protection. This approach offers a flexible and sustainable model for beekeeping that can be adapted to different systems worldwide.

## 1. Introduction

Honey bees are well-known for their essential role in ecosystem conservation, pollinating both cultivated and wild plant species. They contribute substantial economic value to agriculture through honey production and related by products while also supporting biodiversity and global food security [1,2,3,4].

Despite advancements in welfare policies for other species, honey bees have historically been overlooked, with only a few references to managed honey bee welfare [5,6]. Recently, Formato and colleagues [7] identified 243 Honey Bee Welfare Practices (HBWPs), categorising them by their impact on the Five Domains model identified in 2015 by Mellor and Beausoleil [8], with reference to the practices already listed in the BPRACTICES project based on the One Health approach [9,10,11]. Their work introduced 28 new honey bee welfare specific practices, with particular attention paid to honey bee physiology, behaviour, sufferings, the quality of their foraging area, and the mental state domain, to ensure a comprehensive welfare approach for honey bees [7].

The One Welfare framework [12] highlights the interconnectedness of humans, animals, and the environment, recognising their equal importance. When applied to honey bees, this concept allows us to acknowledge the impact of honey bee welfare on ecological and human wellbeing, and vice versa. It considers various aspects, including socio-economic value, environmental protection, pollinator and biodiversity conservation, human and animal rights, soil preservation, food security, and food safety [13,14,15]. A core aspect of this approach is its broad interdisciplinary nature, encompassing fields such as veterinary medicine, law, ethics, economics, sociology, human health, and biology [16], which makes the approach more comprehensive.

Given its broad scope, the One Welfare framework in beekeeping does not place one stakeholder above others, but promotes balanced actions, offering a reasonable compromise to equally benefit humans, bees, and the environment. It also emphasises the importance of mental experiences and individual preferences, in addition to physical health [8,17,18,19]. This way, it is possible to promote a resilient, welfare-centred apiculture model where everyone counts. Gaining a complete understanding of the impacts of welfare practices allows for more informed decision making, providing every type of beekeeping with the opportunity to become welfare-friendly over time. Furthermore, this framework integrates the behavioural, physiological, and mental aspects of honey bee care with the economic, physical, and mental wellbeing of beekeepers. It also considers overall environmental enrichment, ensuring a comprehensive welfare approach. To fully incorporate One Welfare into applied beekeeping, this work is the first to explore the integration of Honey Bee Welfare Practices with human wellbeing and environmental welfare. The aim of this study, in fact, is to obtain a list of One Welfare Practices in Beekeeping, considering all stakeholders as equally important. For that reason, we categorise all practices, considering their impact on productivity and beekeeper time consumption. We also determine whether these practices benefit bees as individuals or as superorganism in the long term. Indeed, bee colonies are considered true “superorganisms”, where individual bees represent “cells”. Therefore, every activity performed by bees should not be seen as the action of a single individual, but rather as a function aimed at the overall operation of the entire superorganism [20]. Given this physiological characteristic of honey bees, we deem it appropriate to consider the potential benefits of each practice either on the individual bee (e.g., the queen) or on the entire colony.

This dual perspective allows us to propose different possible management options to beekeepers, depending on their orientation in beekeeping management (e.g., professional, hobbyist, conventional, organic, size of the beekeeping operation, etc.), thus enabling us to achieve our purpose.

## 2. Materials and Methods

After providing, for the first time, a definition of the One Welfare Practices in Beekeeping, we further reviewed the 243 Honey Bee Welfare Practices (HBWPs) already identified and defined by Formato and colleagues [7], integrating the One Health approach with the One Welfare Framework. This allowed us to focus on welfare and its compromises, not only for honey bees, but also for humans and the environment. We improved the English for all practices, merged some of them, and divided others for easier reading and comprehension. Additionally, some practices were removed to avoid repetition, and 61 new ones were added, reaching a total of 295 (Appendix A). For instance, some of these new practices address phytosanitary product intoxication or poisoning, a topic entirely overlooked in prior research, yet one that commonly affects beekeepers worldwide. After that, we classified them according to their positive impact on the following three “welfare categories”: Human Wellbeing (HW), Environmental Welfare (EW) and Honey Bee Welfare (HBW).

To better understand how to assign the welfare categories to each HBWP, it was important first to know the definitions of human wellbeing and environmental welfare.

There are multiple perspectives from which welfare and its subjects of interest can be framed. For instance, the World Health Organization (WHO) does not provide a single definition of “human welfare or wellbeing”, but often refers to the overall wellbeing and quality of life of individuals, encompassing physical, mental, and social dimensions. Human wellbeing is, in fact, explained by the WHO as “a resource for daily life determined by social, economic and environmental conditions. Wellbeing encompasses quality of life and the ability of people and societies to contribute to the world with a sense of meaning and purpose” [21].

Conversely, the World Organisation for Animal Health (WOAH) defines animal welfare as “the physical and mental state of an animal in relation to the conditions in which it lives and dies” [22].

A good state of welfare, whether for animals or humans, implies that an individual is healthy, comfortable, well-nourished, safe, able to express natural behaviours, and free from unpleasant states such as pain, fear, and distress.

In essence, animal welfare refers to the quality of life of an animal, taking into account its health, wellbeing, and ability to thrive within its environment. The WOAH provides international guidance and standards aimed at promoting animal welfare worldwide.

Instead, the One Welfare Framework explains environmental welfare as, for people, “leading a lifestyle that values the relationship between ourselves, our community, and the environment. Individual wellbeing is affected by the environments we live in and we as individuals also have a significant impact on these environments. Cultivating environmental welfare requires the ability of humans to recognise the responsibility to protect the earth and promote lifestyle practices that serve to sustain the natural environment and its resources” [23].

Therefore, these definitions encompass many aspects and levels of human life, including people’s responsibility to act in a certain way to take good care not only of themselves, but also of the planet and its resources.

Moreover, we decided to analyse all the HBWPs considering their impact on production (P) and time consumption for beekeepers (T), and we deepened the practices’ possibility to have more benefits for the bee as an individual (I) or the superorganism in the long term (S). Then, we assigned each HBWP to positivity (↑), negativity (↓), equality (=), or not applicable (NA) effect indicators, according to the impact that they had on those four variables (P, T, I, and S). In some cases, it was necessary to use an asterisk symbol (*), instead of the indicators already listed, to indicate the need for further studies.

Our judgements on these variables were based on the already validated Good Beekeeping Practices (GBPs) [11] and Biosecurity Measures in Beekeeping (BMBs) [10], also considering the Five Domains Model and previous work on HBWPs [7]. Furthermore, our personal experience in beekeeping was taken into account when assessing these practices. To ensure objective validation, our hope is to later engage a group of experts. We would then compare our assessments and exchange views to formally endorse these One Welfare practices.

We evaluated the time variables of mandatory practices and legal obligations detailed in Appendix A (such as compliance with veterinary regulations and competent authority directives for WOAH-listed diseases) as “not applicable”. This is because beekeepers have no discretion regarding these requirements, therefore, the time needed for their application cannot be evaluated, as compliance is obligatory. Conversely, in instances where a practice might be mandatory only in certain countries (e.g., the registration of the apiary and/or beekeeper in the national registry), we provided an assessment for the variables examined.

## 3. Results

The One Welfare Practices in Beekeeping can be defined as “*all operational and integrative activities intended to promote the best living conditions for honey bees, humans, and the environment, by achieving an optimal welfare balance between the three components*”.

After our revision, we effectively obtained a total of 295 One Welfare Practices (Appendix A) based on their benefits and impacts on Human Wellbeing (HW), Environmental Welfare (EW), and Honey Bee Welfare (HBW). We had to consider 61 new practices [Honey Bee Welfare Practices (Table 1)] involving human, environmental, and honey bee welfare, omitted in the previous work by Formato et al. [7], and we added them to the most suitable existing headings.

While 280 practices positively impact Honey Bee Welfare (13 of which are not in bold, thus considered to have a lower positive impact than others), this may not always be the predominant factor over Environmental or Human Wellbeing effects. In fact, 106 practices enhance Human Wellbeing (HW) (including 50 not in bold), and 34 support Environmental Welfare (EW) (17 of which are not in bold).

Regarding productivity, 213 practices positively affect this (↑), while 5 have a negative impact (↓), 56 are found to have no impact (=), 19 practices are rated as “not applicable” (NA), and in only 2 cases is the assigned impact either positive or negative (↑/↓).

On the other hand, 9 practices are time-saving (↑), 236 have a negative impact because they are time-consuming (↓), 44 have no impact on time (=), for 4 practices we decide it is appropriate to write “not applicable” (NA), and in 2 cases we assign both a negative and a non-applicability rating (↓/NA).

Concerning the benefit of the practices for honey bees as individuals (I), 196 have a positive impact (↑), 17 have a negative one (↓), 28 are “not applicable” (NA), 28 have no impact (=), 25 are rated as both positive and negative (↑/↓), and in 1 case, we use the asterisk symbol (*) due to the need for further studies.

In the case of long-term benefits for the superorganism (S), 255 practices positively affect it (↑), none have a negative impact (↓), 8 practices are considered as “not applicable” (NA), 23 have no impact (=), 8 practices are both positive and negative (↑/↓), and in 1 case, the asterisk symbol “*” is assigned due to the need for further studies (the same case as mentioned before).

## 4. Discussion

Honey Bee Welfare Practices have been increasingly investigated to assess their overall impact on Honey Bee, Human, and Environmental Welfare, with the aim to identify new One Welfare Practices in Beekeeping.

This assessment included considerations of the daily practices’ effects on beekeepers (e.g., time demand), the potential impacts on productivity, and the added value of bee-friendly management. Moreover, benefits were considered both for honey bees (as individuals and/or as a superorganism in the long term) and for beekeepers, recognising that some practices merely or primarily benefit human welfare [e.g., practice n°73: Limit the lifting of weights (e.g., when harvesting supers or when moving hives) and use back support devices if needed].

Our final goal was to provide a balanced perspective in a holistic approach that accounts for the wellbeing of bees, humans, and the environment, offering an objective evaluation of each practice to facilitate reasonable compromises and promote equitable welfare across all ecosystem components.

The results highlight that most of the practices (236) were reported as being time-consuming for beekeepers. Being a beekeeper requires significant commitment, not only to maintain and improve productivity, but also to implement various strategies aimed at enhancing animal care. A clear distinction emerged between professional beekeepers and hobbyists. In fact, some practices, such as monitoring honey bee behaviours (practice n°97), could be unfeasible for professionals due to the large number of colonies in the apiary. In contrast, hobbyists may be more attentive to such practices because of greater dedication, time availability, and often having fewer hives to manage. However, it is important to note that many practices perceived as time-consuming may only be so in the short term, while over time, they could contribute to improving final outcomes in terms of productivity and health. For instance, adhering to hygiene rules and protocols both periodically and when handling dead colonies (practices n°7 and 8) or adopting the queen caging technique in association with acaricide treatments can reduce disease outbreaks and decrease productivity from a long-term perspective. Similarly, prioritising gentle beekeeping methods during hive inspections (practice n°53) reduces the risk of colony stress, increasing the long-term resilience of the bees. These findings underline the need for further studies to better understand the differences between practices that are time-consuming in the short term and those that provide long-term benefits to both bees and beekeepers.

Some practices, while beneficial for beekeepers, may conflict with honey bee welfare and vice versa, requiring compromises. For example, not marking the queen may benefit her welfare by sparing unnecessary suffering, but can be time-consuming for beekeepers to find her during hive inspections if not marked, to the point of being considered unfeasible for some professional beekeepers. Additionally, this can lead to a loss of beekeeper welfare, resulting in longer workdays and an increased risk of bee mismanagement. For example, bees might be exposed to cold or rain in adverse weather, or the colony could become orphaned if the queen, after being marked and released, is not recognised by workers due to a different odour, potentially leading to her death. Similarly, not marking the queen can lead to failure or wasting time in locating her on the frames, especially for inexperienced beekeepers. Given these considerations, a more feasible compromise might be only marking queens of high genetic value. Of course, marking queens causes stress to the individual bee, but from a long-term perspective, it may be time-saving for beekeepers and provide benefits to the whole colony. The challenge lies in deciding whether to mark the queen or not, as there are advantages and disadvantages with both options.

When considering queen substitution (practice n°76), replacing the queen offers potential benefits for production and can positively influence the superorganism’s welfare in the long run. Nevertheless, the initial phase can negatively impact bees, causing stress due to the loss of the queen and her pheromone. Moreover, queen substitution directly leads to distress for the individual bee.

On the other hand, allowing an older queen to remain can result in a decline in production over time, which affects commercial beekeepers. This highlights the need for a balanced approach. For instance, replacing the queen every 2–3 years rather than every year, or only once a certain threshold of production decline is reached, could be a viable strategy. Therefore, it is important to thoroughly evaluate the queen’s health and productivity before replacing her, as she might be capable of sustaining a strong and healthy colony for more than two or three years. As a matter of fact, the latter option may be more feasible for beekeepers attentive to bee care.

Under the heading “General Apiary Management” within the “colony management” topic, seven practices (77, 78, and 80–84) are linked to swarm prevention. We assessed these practices as having a primarily positive impact on Human Wellbeing (HW) rather than Honey Bee Welfare (HBW). This is because swarming is a natural, physiological characteristic of honey bees, and preventing it could impede their natural behaviour, potentially conflicting with definitions of animal welfare [24,25]. However, if swarming occurs and the beekeeper does not adopt proper biosecurity measures to control *Varroa destructor*, this could negatively affect honey bee welfare. For other variables (P, T, I, and S), we assigned a positive impact on productivity due to the avoided loss in live honey bees; regarding time consumption, preventing swarming is certainly a challenging and time-consuming task for beekeepers. Still, collecting natural swarms can be equally demanding, with added risks to human health from falls or stings during collection. Also, we assigned an either positive or negative judgement to individual and superorganism welfare. In fact, while there is a negative physiological impact, it is possible to distract bees from breeding through specific procedures, which can mitigate negative welfare outcomes.

Furthermore, practice n°78, “prevent swarming by performing colony split”, offers a beneficial solution that satisfies both the beekeeper’s and the honey bees’ needs. This involves adopting an “artificial swarming” method by splitting colonies to create new ones [26].

Despite prevention efforts, swarming is not always guaranteed to be avoided, as the colony might still swarm. For this reason, selecting apiary sites with shrubs (practice n°44) makes it easier for beekeepers to recover swarms. This is particularly important when establishing a new apiary or relocating an existing one.

Other important welfare practices in beekeeping involve taking samples for laboratory analyses (practices n°100, 137, 186, 187, 202, and 206). These analyses can be crucial for monitoring and controlling overall disease situations within the apiary, as well as assessing environmental quality. Although relevant, this operation may negatively impact individual bees, especially when disruptive samples (often involving live bees) are collected. For this reason, it is better to choose non-invasive samples whenever possible, such as hive debris, powdered sugar, wax, pollen, propolis, and honey from combs. The INSIGNIA-EU project exemplifies this approach, using non-disruptive methos to monitor environmental pollution while prioritising honey bee welfare [27]. However, while sampling for in-depth analytical studies is critical for the potential survival of the entire colony and human health, it can also support management efforts by providing long-term benefits to the superorganism or the environment.

The purpose of using a queen excluder (as outlined in practices n°88, 235, 246) is to prevent the queen and brood diseases from entering the honey supers, thereby ensuring a cleaner, purer, and safer product. While this practice benefits honey quality and honey bee health, it can be costly and time-consuming, potentially impacting professional beekeepers in a negative way.

Confining the queen within the brood chamber positively affects honey production quality, but may be stressful for the queen herself. Nevertheless, this approach is valuable for guaranteeing honey quality and reducing the spread of infectious diseases. Consequently, we assigned a negative impact (↓) to the individual bee variable (I).

Conversely, since worker bees can freely pass through the excluder to collect nectar in the honey supers, while the queen cannot, this results in the absence of brood diseases (e.g., AFB, EFB, SHB, virosis, etc.), ensuring better health of the superorganism in the long term. Therefore, the impact assigned to the superorganism variable (S) was positive (↑).

Similarly, implementing a brood interruption prior to *Varroa* spp. treatment (practices n°46, 161, 169, 182) is an excellent strategy not only to maximise treatment efficacy, but also to promote the long-term welfare and health of the superorganism by controlling *Varroa* spp. infestation levels. However, we assigned a negative impact to the individual bee variable (I) due to the potential stress and mortality risks associated with queen caging, which can result from handling or prolonged confinement. Additionally, this practice is time-consuming for beekeepers and carries the risk of bees failing to recognise the queen post-caging, leading to her potential death.

## 5. Conclusions

Through the One Welfare approach adopted in this study, we present a comprehensive overview of the potential impacts of beekeeping practices on honey bee, human, and environmental welfare. For instance, practices n°27, 28 and 54, which take into account the communication between local stakeholders (beekeepers, farmers, advisors, and veterinarians) to coordinate actions for preventing and controlling diseases, minimise the risk of intoxication from PPPs, and coordinate efforts for managing foraging resources, embrace all three welfare categories (HBW, HW, and EW) due to the fact that all components might benefits from this approach. This approach promotes a resilient, welfare-centred model for apiculture, where all stakeholders are considered and beekeepers can make informed decisions about hive management based on the variables they prioritise.

At the core of this methodology lies the concept of compromise, which is central to the dynamic balance advocated by One Welfare. This balance becomes evident when beekeepers, weighing various economic factors and the importance of honey bee welfare, as outlined in these practices, determine their apiary management strategies.

Our study resulted in a list of “One Welfare practices in beekeeping” that supports a holistic approach to welfare. This might represent a versatile framework applicable to other managed pollinators, such as bumblebees or insects in general, and agricultural practices after more specific research and further studies, thereby broadening the scope of the One Welfare approach and extending its comprehensive perspective beyond honey bees.

For future validation of our outcomes, we aim to organise an international expert elicitation meeting.

## Figures and Tables

**Table 1:** New Honey Bee Welfare Practices [7], modified], within the One Welfare approach.

Honey Bee Welfare Practice	Welfare Category
Avoid areas where toxic (e.g., with pyrrolizidine alkaloids) plants (e.g., *Echium* spp., *Eupatorium* spp. and *Senecio* spp.) can be found in a significant quantity	**HW**
Limit the lifting of weights (e.g., when harvesting supers or when moving hives) and use back support devices if needed	**HW**
Avoid areas where allergenic plants (e.g., *Ambrosia trifida* and *Artemisia vulgaris*) can be found in a significant quantity	**HW**
In the case of abnormal mortality, contact veterinary services and the competent authorities	**HBW**, EW
Be mindful of any unusual odour when opening the hive—a foul smell of carpentry glue is commonly associated with suspected cases of the clinical form of AFB	**HBW**
In the case of unplanned phytosanitary treatments (with products toxic to bees), the farmer should immediately notify the beekeeper to assess whether to move the hives as soon as possible or close them before the morning flight begins by covering them with wet cloths, supplying them with water, and ensuring air circulation	**HBW**, EW
In the case of poisoning caused by phytosanitary treatments (with products toxic to bees), the beekeeper should move the hives as soon as possible, if feasible, or close them before the morning flight begins by covering them with wet cloths, supplying them with water, and ensuring air circulation	**HBW**
In the case of unplanned phytosanitary treatments or poisoning, replace the queen (if old) with a younger, more vigorous one	**HBW**
In the case of slow and progressive mortality with contaminated pollen, remove the combs and replace them with others containing pollen from healthy colonies, or provide protein nutrition in addition to sugar	**HBW**
Carry out inspections for honey bee diseases before transferring colonies to a new location	**HBW**
Follow national rules for migratory beekeeping	**HBW**, **HW**, **EW**
Have enough space for storage rooms/working tools	**HBW**, **HW**
Inspect colonies during suitable weather conditions to reduce stress	**HBW**, **HW**
Provide hives with a proper stand	**HBW**
Guarantee proper space between hives	**HBW**
Avoid placing apiaries in areas exposed to excessive humidity	**HBW**
Avoid obstacles for bees (e.g., high grass/snow in front of the hive entrance)	**HBW**
Ensure the presence of trees (or other barriers) to create protection against weather stressors (e.g., heat, wind, etc.)	**HBW**
Prevent the theft of hives (e.g., assess presence/build a fence around the apiary)	**HW**
Seasonally relocate the apiary, where possible (e.g., taking into account the season, wintering near warmer areas)	**HBW**
Keep a safe distance from houses/villages (for human safety)	**HW**, **HBW**
Provide a proper orientation of the hive entrances so that the sun can reach them from the early morning hours to sunset	**HBW**
Choose the best colonies as larvae donors	**HBW**, **HW**
Do not keep diseased colonies: intervene properly if you suspect or detect a disease to avoid the spread of pathogens (e.g., sampling for laboratory analysis; treatment; destruction, etc.)	**HBW**, **HW**
Evaluate colony food stores, considering the availability of honey and pollen and the floral source of honey	**HBW**
Evaluate the space needed by the colony, according to its strength	**HBW**
Evaluate the mating success of the queen	**HBW**, **HW**
Verify that the recently mated queen has started oviposition	**HBW**, **HW**
Expand the colony to give it space	**HBW**
Assess colony strength during hive inspection	**HBW**
During hive inspections, verify the presence of the queen	**HBW**
During hive inspections, verify oviposition activity of the queen	**HBW**
During hive inspections, assess signs of diseases	**HBW**
Practice the proper overwintering of hives	**HBW**
Properly process beeswax for the production of new wax foundations	**HBW**
Perform wax moth control	**HBW**
Adopt proper comb storage	**HBW**
Replace old beehive frames (when degraded, spent, or mouldy to ensure hygiene standards and to maintain space for oviposition)	**HBW**
Replace queens with poor hygienic behaviour	**HBW**
Identify the best moment for Varroa treatments according to the national climatic areas	**HBW**, **EW**
Properly use formic acid for Varroa treatment	**HBW**, **EW**
Properly use oxalic acid (including sublimated) for Varroa treatment	**HBW**, **EW**
Properly use thymol (e.g., applying only when environmental temperature is high enough)	**HBW**, **EW**
Properly use other registered medical products with a low environmental impact	**HBW**, **EW**
Do not move potentially AFB-infected materials (e.g., frames/bees/feeds) to healthy colonies	**HBW**
Ensure the prompt notification of American Foulbrood (AFB) cases to adjacent beekeepers	**HBW**, **HW**
Establish a group of advisors in the local beekeeping community to help beekeepers in managing the spread of diseases	**HBW**
Inform your neighbours about EFB at your apiary	**HBW**, **HW**
Do not move potentially EFB-infected materials (e.g., frames/bees/feeds) to healthy colonies	**HBW**
Use only feed safe for bees (e.g., specifically registered, without bee pathogens, etc.)	**HBW**
Clean feeders after their use	**HBW**
If robbing occurs, temporarily close the hive or reduce the entrances, clean honey residues from hives surfaces or surrounding areas, and use the smoker	**HBW**
Do not heat sugar solutions over tap-warm temperature	**HBW**
Use proper feeders	**HBW**, **HW**
Keep records of harvesting data	**HW**
Use a recording method (physical or digital registers/apps) that fits to your needs and the size of your beekeeping operation	**HW**, **HBW**
During hive inspections, record the characteristics of honey bee queens (e.g., brood pattern, amount of eggs laid, cohesiveness of the colony, etc.)	**HBW**, **HW**
During hive inspections, record any zootechnical performance of the colony (productivity, hygienic behaviour, docility, etc.)	**HBW**, **HW**
Mark/identify hives that need subsequent attention	**HBW**, **HW**
Keep records of the veterinary medicinal products authorised in your country	**HBW**, **HW**
Periodically update the list of the authorised veterinary medicinal products	**HBW**, **HW**

## Data Availability

Data is contained within the article and Appendix A.

## Abbreviations

The following abbreviations are used in this manuscript:

HBWPs	Honey Bee Welfare Practices
HW	Human Wellbeing
EW	Environmental Welfare
HBW	Honey Bee Welfare
WHO	World Health Organization
WOAH	World Organization for Animal Health
P	Production
T	Time consumption
I	Benefit for the individual bee
S	Benefit for the superorganism in the long term
AFB	American Foulbrood
EFB	European Foulbrood
SHB	Small Hive Beatle
PPPs	Plant Protection Products (phytosanitary products)
NaOH	Sodium hydroxide
NaClO	Sodium hypochlorite
ABPV	Acute Bee Paralysis Virus
DWV	Deformed Wing Virus
IAPV	Israeli Acute Paralysis Virus
KBV	Kashmir Bee Virus
SBV	Slow Bee Paralysis Virus
CO2	Carbon dioxide
P. larvae	Paenibacillus larvae
PCR	Polymerase Chain Reaction
M. plutonius	Melissococcus Plutonius
A. tumida	Aethina tumida

## Appendix A. One Welfare Practices in Beekeeping

**Table A1:** HBW: honey bee welfare; HW: human wellbeing; EW: environmental welfare; Bold letters in welfare category: greater positive impact; P: productivity impact; T: time consumption for beekeepers; I: benefit for the individual bee; S: benefit for the superorganism in the long term; ↑: positive impact; ↓: negative impact; =: no impact; NA: not applicable; *: need for further studies. The underlined practices are the five new ones.

Heading	Honey Bee Welfare Practice	Welfare Category	P	T	I	S
**1. GENERAL APIARY** **MANAGEMENT** Transportation	**1.** Comply with legal obligations concerning restrictions on animal movements in the case of listed diseases	**HBW**	↑	NA	↑	↑
	**2.** Carry out inspections for honey bee diseases before transferring colonies to a new location	**HBW**	↑	↓	↑	↑
	**3.** Transport/move only healthy colonies	**HBW**	↑	=	↑	↑
	**4.** Follow national rules for migratory beekeeping	**HBW**, HW, EW	NA	NA	NA	NA
	**5.** Avoid transporting hives during the warmer hours of the day. Ensure proper ventilation by providing adequate openings for airflow within the hives and watering	**HBW**	↑	↓	↑	↑
	**6.** To minimize physical stress during transport, schedule regular stopovers to allow the bees to rest and recover	**HBW**	↑	↓	↑	↑
**1. GENERAL APIARY** **MANAGEMENT** Hygiene	**7.** Respect hygiene rules (e.g., periodically clean suits, gloves, etc.)	**HBW**	↑	↓	↑	↑
	**8.** Follow proper hygiene procedures when handling dead colonies (combs, food stores, boxes, etc.)	**HBW**	↑	↓	↑	↑
	**9.** After inspection of hives affected by transmissible diseases, disinfect levers and other potentially contaminated equipment (e.g., gloves)	**HBW**	↑	↓	↑	↑
	**10.** Do not place honey supers directly on the ground (avoid contamination with *Clostridium Botulinum*)	**HW**	=	↓	=	=
	**11.** Avoid exposure to dust during the transport of supers from the apiary to the honey processing facility	**HW**	=	=	=	=
	**12.** Do not place beehives directly on the ground	**HW**	=	=	=	=
	**13.** Wear disposable gloves when handling diseased hives	**HBW**	=	=	↑	↑
**1. GENERAL APIARY** **MANAGEMENT** Bee Health	**14.** Use only bees and brood combs from healthy colonies for nuclei	**HBW**	↑	=	=	↑
	**15.** Have enough space for storage rooms/working tools	**HBW**, HW	=	↓	=	=
	**16.** Balance colony strength by transferring frames only between healthy hives	**HBW**	↑	=	↑	↑
	**17.** Inspect colonies during suitable weather conditions to reduce stress	**HBW**, HW	↑	=	↑	↑
	**18.** Buy new hives only after thorough inspection for bee diseases, preferably with a health certificate from a veterinarian	**HBW**	↑	=	↑	↑
	**19.** Keep only healthy colonies in the apiary	**HW**, HBW	↑	↓	=	↑
	**20.** In case of abnormal bee mortality, contact veterinary services and the competent authorities	**HBW**, EW, HW	↑/↓	↓	↑	↑
	**21.** Avoid placing apiaries in areas with environmental pollutants or place them in areas with minimal exposure (heavy metals, phytosanitary products, pesticides etc.)	**HW**, **HBW**, **EW**	↑	↓	↑	↑
	**22.** When equalising hives, maintain a proper balance between nurse bees and brood; preferably use combs with emerging bees to fortify weak colonies	**HBW**, HW	↑	↓	=	↑
	**23.** Prefer queens that are disease-resistant and well adapted to local climatic conditions	**HBW**, HW	↑	↓	↑	↑
	**24.** Keep newly introduced colonies separate from the existing stock for an appropriate period (at least one month) to monitor them against diseases and prevent transmissions (quarantine apiary)	**HBW**	↑	↓	↑	↑
	**25.** Avoid introducing swarms of unknown origin, or colonies or queens from other apiaries, whenever possible	**HBW**	=	=	↑	↑
	**26.** Keep weak colonies in a quarantine apiary	**HBW**	=	↓	↑	↑
	**27.** Communicate with local stakeholders (e.g., beekeepers, farmers, advisors, veterinarians) to coordinate actions in preventing and controlling diseases (such as varroosis)	**HBW**, **HW**, **EW**	↑	↓	↑	↑
	**28.** Communicate with local stakeholders (e.g., beekeepers, farmers, advisors, veterinarians) to minimise the risk of intoxication from PPPs	**HBW**, **HW**, **EW**	↑	↓	↑	↑
	**29.** Adopt effective techniques to control honey bee predators (such as ants, wasps, bee-eaters, rats, and bears)	**HBW**, HW	↑	↓	↑	↑
	**30.** Ensure access to expert support (e.g., veterinarians, technicians, etc.) for assistance when needed	**HBW**	↑	=	↑	↑
**1. GENERAL APIARY** **MANAGEMENT** Apiary Management	**31.** Keep an appropriate number of hives (e.g., consider the amount of melliferous plants and sources of water available in the area where the apiary is located)	**HBW**	↑	↓	↑	↑
	**32.** Avoid leaving beekeeping material abandoned in the apiary	**HBW**	↑	=	↑	↑
	**33.** Maintain a proper balance between the number of hives and the amount of melliferous plants/pollen sources in the area where the apiary is located	**HBW**, HW, EW	↑	↓	↑	↑
	**34.** Provide hives with a proper stand	**HBW**	↑	=	↑	↑
	**35.** Guarantee proper space between hives	**HBW**	↑	=	↑	↑
	**36.** Avoid placing apiaries in areas exposed to excessive humidity	**HBW**	↑	↓	↑	↑
	**37.** Avoid placing apiaries in areas exposed to excessive wind	**HBW**	↑	↓	↑	↑
	**38.** Choose locations for placing apiaries that are not too sunny in summer or too shady in winter	**HBW**, HW	↑	↓	↑	↑
	**39.** Adjust the number of hives in the apiary based on season and the availability of pollen, nectar, and honey dew resources	**HBW**, EW, HW	↑	↓	↑	↑
	**40.** Set up apiaries on a firm ground	**HW**	=	↑	=	=
	**41.** Prevent colony drift by avoiding placing too many colonies in a single row	**HBW**, HW	↑	=	↑	↑
	**42.** Set up apiaries in an area accessible to vehicles	**HW**, **HBW**	↑	↑	↑	↑
	**43.** Prevent robbing by ensuring that hives are well maintained, with no broken parts or openings	**HBW**, HW	↑	↓	↑	↑
	**44.** Place hives in biodiverse areas with a variety of melliferous and nectariferous plants, as well as shrubs (that offer natural foraging and swarming opportunities for honey bees)	**HBW**	↑	↓	↑	↑
	**45.** Position the hive in an area exposed to minimal light pollution and excessive vibrations	**HBW**	↑	↓	↑	↑
	**46.** In the case of brood interruption, confine the queen on comb, allowing her to move freely and lay eggs	**HBW**, HW	↑	↓	↑/↓	↑
	**47.** Maintain the correct bee space between combs (e.g., removing any abnormal comb constructions)	**HBW**, HW	↑	↓	↑	↑
	**48.** Breed your own autochthonous, locally adapted queens	**HBW**, HW, EW	↑	↓	↑	↑
	**49.** Avoid performing artificial insemination on the queen bee	**HBW**	=	↑	↑	=
	**50.** Perform genetic selection of the honey bees	**HBW**	↑	↑	↑/↓	↑
	**51.** Avoid relocating hives unless strictly necessary	**HBW**	↑	↑	↑	↑
	**52.** Avoid rearranging the order of combs unless necessary	**HBW**	↑	↑	↑	↑
	**53.** Prioritise gentle beekeeping practices during inspections to minimise stress and harm (e.g., by avoiding abrupt or sudden movements, using well-fitted latex gloves, lifting or moving combs/frames carefully, avoiding bee crushing when closing the hive top, and using the smoker properly)	**HBW**, **HW**	↑	=	↑	↑
	**54.** Communicate with other local stakeholders (e.g., beekeepers, farmers, advisors, veterinarians) to coordinate efforts in managing foraging resources	**HBW**, **HW**, **EW**	↑	↓	↑	↑
	**55.** Avoid obstacles for the bees (e.g., high grass/snow in front of the hive entrance)	**HBW**	↑	↓	↑	↑
	**56.** Ensure the presence of trees (or other barriers) to create protection against weather stressors (e.g., heat, wind, etc.)	**HBW**	↑	↓	↑	↑
	**57.** Prevent the theft of hives (e.g., assess presence/build a fence around the apiary)	**HW**	↑	↓	=	=
	**58.** Seasonally relocate the apiary, where possible (e.g., taking into account the season; wintering near warmer areas)	**HBW**	↑	↓	↑	↑
	**59.** Listen the sounds coming from the hive before opening it	**HBW**, HW	=	↓	↑	↑
**1. GENERAL APIARY** **MANAGEMENT** Wintering	**60.** (Before winter) reduce the empty space in the hive	**HBW**	↑	↓	↑	↑
	**61.** Reduce the size of the hive entrance	**HBW**	↑	↓	↑	↑
	**62.** Perform beehive box maintenance (replacing parts or painting; verify the integrity of hive boxes, if needed)	**HBW**	↑	↓	=	↑
	**63.** Check the positioning of the frames containing stores to ensure they are accessible to the cluster	**HBW**	↑	↓	↑	↑
	**64.** Reduce the number of frames in the hive box	**HBW**	↑	↓	↑	↑
	**65.** Insert a divider board to reduce the volume of the hive nest	**HBW**	↑	↓	↑	↑
	**66.** Wrap the hive in black tar paper, if needed	**HBW**	=	↓	↑	↑
	**67.** Ensure honey bees are housed under optimal physiological conditions, with proper temperature and humidity control	**HBW**	↑	↓	↑	↑
**1. GENERAL APIARY** **MANAGEMENT** Human Health	**68.** Wear personal protective clothing and equipment when visiting honey bee colonies	**HW**, **HBW**	↑	↓	↑	↑
	**69.** Avoid areas where allergenic plants (e.g., *Ambrosia trifida* and *Artemisia vulgaris*) can be found in a significant quantity	**HW**	=	↓	=	=
	**70.** Avoid areas where toxic (e.g., with pyrrolizidine alkaloids) plants (e.g., *Senecio* spp., *Eupatorium* spp. and *Echium* spp.) can be found in a significant quantity	**HW**	=	↓	*	*
	**71.** Keep corticosteroids or other appropriate medicines ready to use during apiary inspections to guarantee the health of operators (for example, in case of anaphylaxis)	**HW**	=	↑	=	=
	**72.** Keep safe distance from houses/villages (for human safety)	**HW**, HBW	=	=	↑	↑
	**73.** Limit the lifting of weights (e.g., when harvesting supers or when moving hives) and use back support devices if needed	**HW**	=	=	=	=
**1. GENERAL APIARY MANAGEMENT** Colony management	**74.** Practice hive management according to region, season, and strength of the colony	**HBW**	↑	=	↑	↑
	**75.** Provide a proper orientation of the hive entrances so that sun can reach them from the early morning hours to sunset	**HBW**	↑	=	↑	↑
	**76.** Replace the queen at least every two or three years to maintain a strong and healthy colony (unless it has high genetic value)	**HBW**, HW	↑	↓	↑/↓	↑
	**77.** Prevent swarming by adding new wax foundations	**HW**, HBW	↑	↓	↑/↓	=
	**78.** Prevent swarming by performing colony split	**HW**, HBW	↑	↓	↑/↓	↑/↓
	**79.** Comply with the scheduled plan for regular beehive inspections	**HBW**, **HW**	↑	↑	↑	↑
	**80.** Prevent swarming by adding honey supers to the hive	**HW**, HBW	↑	↓	↑/↓	=
	**81.** Prevent swarming by removing the entrance reducer	**HW**, HBW	↑	↓	↑/↓	=
	**82.** Prevent swarming by adopting genetic selection of the queens	**HW**, HBW	↑	↓	↑/↓	=
	**83.** Prevent swarming by inserting drawn combs into the hive	**HW**, HBW	↑	↓	↑/↓	=
	**84.** Prevent swarming by removing the beehive’s bottom board	**HW**, HBW	↑	↓	↑/↓	=
	**85.** Mark the queen with a colour code corresponding to her year of birth	**HW**, HBW	=	↓	↓	↑
	**86.** Explore alternative methods for identifying the queen without marking her	HBW, **HW**	=	↓	↓/↑	↑
	**87.** Orientate the hive entrance so that the sun can reach the bees in the early morning hours	**HBW**	↑	=	↑	↑
	**88.** Use a queen excluder	HW, **HBW**	↑	↓	↓	↑
	**89.** Prevent drift by marking the front and entrance of the hive with numbers or identification signs	**HBW**, HW	↑	↓	↑	↑
	**90.** Indicate the age of the combs on the top bar of the frame (e.g., the year of placement of the frame with foundation)	**HW**, HBW	↑	↓	↑	↑
	**91.** Provide adequate openings in the hive for air flow, if needed	**HBW**	↑	↓	↑	↑
	**92.** Reduce the opening of the hive entrance during robbing and cold periods and increase the opening of the hive entrance during the hot season	**HBW**	↑	↓	↑	↑
	**93.** Encourage the natural construction of wax combs in the spring	**HBW**	↓/↑	↓	↑	↑
	**94.** If bees show signs of agitation, restlessness or aggression, wait for them to calm down before proceeding with inspections	**HW**, **HBW**	=	↓	↑	↑
	**95.** Place apiaries at a lower height than the foraging area, allowing foragers carrying nectar and pollen to return to their hive without excessive fatigue	**HBW**	↑	↓	↑	↑
	**96.** Do not open the hive at night or when it is rainy or windy	**HBW**, HW	=	=	↑	↑
	**97.** Monitor honey bee behaviours, including flight activity, noise levels, aggression, calmness, and other behavioural cues	**HBW**	=	↓	↑	↑
	**98.** Maintain appropriate distances between different apiaries to avoid spread of diseases	**HBW**	↑	=	↑	↑
	**99.** Choose the best colonies as larvae donors	**HBW**, HW	↑	↓	↑	↑
	**100.** Do not keep diseased colonies: intervene properly if you suspect or detect a disease to avoid the spread of pathogens (e.g., sampling for laboratory analysis; treatment; destruction, etc.)	**HBW**, HW	↑	↓	↓/↑	↓/↑
	**101.** Evaluate colony food stores, considering the availability of honey and pollen and the floral source of honey	**HBW**	=	↓	↑	↑
	**102.** Evaluate the space needed by the colony, according to its strength	**HBW**	↑	↓	↑	↑
	**103.** Evaluation of mating success of the queen	**HBW**, HW	↑	↓	↑	↑
	**104.** Verify that the recently mated queen has started oviposition	**HBW**, HW	↑	↓	↑	↑
	**105.** Expand the colony to give it space	**HBW**	↑	↓	↑	↑
	**106.** Assess colony strength during hive inspection	**HBW**	↑	↓	↑	↑
	**107.** During hive inspections, verify the presence of the queen	**HBW**	↑	↓	↑	↑
	**108.** During hive inspections, verify the oviposition activity of the queen	**HBW**	↑	↓	↑	↑
	**109.** During hive inspections, assess signs of diseases	**HBW**	↑	↓	↑	↑
	**110.** Optimise inspection time/number of visits (e.g., to minimise stress) by planning an inspection schedule	**HBW**, **HW**	↑	↑	↑	↑
	**111.** Practise the proper overwintering of hives	**HBW**	=	↓	↑	↑
	**112.** Perform selection to obtain queens that are more resistant to disease and adapted to local climatic conditions	**HBW**, HW	↑	↓	↑	↑
	**113.** Properly process beeswax for the production of new wax foundations	**HBW**	↑	↓	↑	↑
	**114.** Perform wax moth control	**HBW**	↑	↓	↑	↑
	**115.** Adopt proper comb storage	**HBW**	↑	↓	↑	↑
	**116.** Replace old beehive frames (when degraded, spent, or mouldy to ensure hygiene standards and maintain space for oviposition)	**HBW**	=	↓	↑	↑
	**117.** Replace queens with poor hygienic behaviour	**HBW**	↑	↓	↓	↑
	**118.** Ensure proper hive density per area	**HBW**, EW	↑	↓	↑	↑
**2. VETERINARY MEDICINES**	**119.** Use only veterinary medicines for honey bees registered in your country or medicines legally imported	**HBW**, HW, EW	↑	=	↑	↑
	**120.** Keep recordings of veterinary medicinal treatments	**HW**	=	↓	=	↑
	**121.** Observe the withdrawal period of veterinary products and ensure that products from treated hives are not used for human consumption until the withdrawal periods have elapsed	**HW**, **EW**	=	↓	=	=
	**122.** When using instruments for applying medicines (such as formic acid dispensers or oxalic acid sublimators), ensure they are appropriate for the task and correctly calibrated for accurate administration	**HBW**, HW	↑	↓	↑	↑
	**123.** Respect the required storage conditions for veterinary medicines and feeds	**HBW**	=	=	↑	↑
	**124.** Dispose of used instrument and devices in a biosecure manner	**HBW**, HW, EW	=	↓	↑	↑
	**125.** Ensure that all treatments or procedures are carried out correctly, following the instructions regarding dosage and application method	**HBW**, HW, EW	=	↓	↑	↑
	**126.** Do not carry out illegal treatments	**HBW**, HW, EW	↑	=	↑	↑
**3. DISEASE AND INTOXICATION** **MANAGEMENT**	**127.** In the case of listed diseases, follow the instructions provided by veterinary regulations and competent authorities	**HBW**	NA	NA	NA	NA
	**128.** In the case of infectious diseases, clean all beekeeping material between uses (e.g., hive bodies, hive bottom boards, feeders, hive tools)	**HBW**	↑	↓	↑	↑
	**129.** In the case of infectious diseases, clean or disinfect the hive box before installing new colonies	**HBW**	↑	↓	↑	↑
	**130.** In the case of unplanned phytosanitary treatments (with products toxic to bees), the farmer should immediately notify the beekeeper to assess whether to move the hives as soon as possible or close them before the morning flight begins by covering them with wet cloths, supplying them with water, and ensuring air circulation	**HBW**, EW	↑	↓	↑	↑
	**131.** In the case of poisoning caused by phytosanitary treatments (with products toxic to bees), the beekeeper should move the hives as soon as possible, if feasible, or close them before the morning flight begins by covering them with wet cloths, supplying them with water, and ensuring air circulation	**HBW**	↑	↓	↑	↑
	**132.** In the case of unplanned phytosanitary treatments or poisoning, replace the queen (if old) with a younger, more vigorous one	**HBW**	↑	↓	↓	↑
	**133.** In the case of slow and progressive mortality with contaminated pollen, remove the combs and replace them with others containing pollen from healthy colonies, or provide protein nutrition in addition to sugar	**HBW**	↑	↓	↑	↑
	**134.** Conduct comprehensive inspections in spring to check for clinical signs of bee diseases and confirm the presence of the queen	**HBW**	↑	↓	↑	↑
	**135.** Conduct comprehensive inspections at the end of the beekeeping season to check for clinical signs of bee diseases and confirm the presence of the queen	**HBW**	=	↓	↑	↑
	**136.** Quickly remove beehives containing dead colonies	**HBW**, HW	↑	↓	NA	NA
	**137.** Take samples for laboratory analyses when sick or dead bees are found, if necessary	**HBW**, **EW**	NA	↓	↓	↑
	**138.** Regularly clean equipment and scrape off wax and propolis	**HBW**	↑	↓	↑	↑
	**139.** Remove and process the wax from all combs of dead or affected colonies	**HBW**	↑	↓	NA	NA
	**140.** Record the health status of the colonies, including diseased or infected colonies. For these, include dates, diagnoses, the IDs of affected colonies, treatments, and results	**HBW,** HW	=	↓	=	↑
	**141.** Replace 30% of hive combs annually	**HBW**, HW	↑	↓	↑	↑
	**142.** Promptly verify any signs of disease, consulting a veterinarian (or a specialist)	**HBW**	↑	↓	↑	↑
	**143.** Do not move frames or any kind of biological material (for example, to balance hives) from one hive to another if their health status is not well known	**HBW**	↑	=	↑	↑
	**144.** Inspect diseased hives only after completing inspections of healthy hives	**HBW**	↑	=	↑	↑
	**145.** Select the best-performing honey bee colonies, based on calmness, productivity, health, strength, honeycomb maintenance by the colony, etc.	**HW**	↑	↓	NA	NA
	**146.** Burn dead colonies	**HBW**	=	↓	NA	NA
	**147.** Remove queens of colonies showing clinical signs of honey bee diseases (EFB, varroa, nosema, chalkbrood, etc.) from selection	**HBW**	↑	↓	↓	↑
	**148.** Try to select and breed colonies that are more disease-tolerant/resistant	**HBW**	↑	↓	↑/↓	↑
	**149.** Record the origin and use of all disinfectants and consumables (all cleaning and disinfection procedures for equipment, including data sheets for each detergent or disinfectant used)	**HBW**, **HW**, **EW**	=	↓	=	↑
	**150.** Keep records that show every time the procedures described above have been effectively implemented (task sheets, self-inspection checks on the effectiveness of the operations)	**HBW**, **HW**, **EW**	=	↓	=	↑
	**151.** Regularly disinfect equipment (e.g., with NaOH, NaClO)	**HBW**, HW	↑	↓	↑	↑
	**152.** Conduct thorough inspections for clinical signs of bee diseases and queen presence before adding supers to the hives	**HBW**, HW	↑	↓	↑/↓	↑
	**153.** When necessary, follow the proper protocol to euthanise bees (whether there is), minimising their suffering	**HBW**	NA	↓/NA	NA	NA
**4. HYGIENE**	**154.** Use torching (blue flame) as a disinfection method for hive boxes and beekeeping tools in the case of transmissible diseases	**HBW**	↑	↓	NA	↑
	**155.** Use bleaching agents (NaOH, NaClO, etc.) as a disinfection method for hives and beekeeping tools in the case of transmissible diseases	**HBW**	↑	↓	NA	↑
	**156.** Perform the incineration of affected colonies, if needed and/or requested (mandatory), in the case of transmissible disease, minimising their suffering	**HBW**	NA	↓/NA	NA	NA
	**157.** Use high-pressure, heated (90 °C) water as a disinfection method for hives and beekeeping tools in the case of transmissible diseases	**HBW**	↑	↓	NA	↑
	**158.** Use autoclaving as a method for the disinfection of hives and beekeeping tools in the case of transmissible diseases	**HBW**	↑	↓	NA	↑
	**159.** Use gamma irradiation as a method for the disinfection of beekeeping tools in the case of transmissible diseases	**HBW**	↑	↓	NA	↑
**5. BIOSECURITY MEASURES** Varroosis	**160.** Always treat varroosis in accordance with national legislation and registration	**HBW**, EW	↑	=	↑	↑
	**161.** Properly use biotechnological methods (e.g., drone brood removal; brood interruption by confining the queen using cages or trapping comb. The latter should be preferred as allows the queen to move more freely and lay eggs)	**HBW**	↑	↓	↓	↑
	**162.** Identify the best moment for Varroa treatments according to the national climatic areas	**HBW**, **EW**	↑	=	↑	↑
	**163.** Adopt/provide hives with screened bottom boards	**HBW**	↑	=	↑	↑
	**164.** Nuclei and swarms should come from colonies with no clinical signs of Varroa-related diseases (ABPV, DWV, IAPV, KBV, SBV, etc.)	**HBW**	↑	=	↑	↑
	**165.** Treat according to an Integrated Pest Management (IPM) concept	**HBW**, **EW**	↑	↓	↑	↑
	**166.** Keep *Varroa* spp. levels in each colony below the harmful threshold	**HBW**	↑	↓	↑	↑
	**167.** Carry out assessment of *Varroa* spp. infestation level (e.g., icing sugar method, CO_2_ test, mite fall, etc.) throughout the year (e.g., in spring at the beginning of the beekeeping season or before harvesting) and after acaricide treatments	**HBW**	↑	↓	↓	↑
	**168.** Treat all colonies of the apiary and those in the same area simultaneously	**HBW**, **EW**	↑	↓	↑	↑
	**169.** Prepare colonies (e.g., absence of brood) before treatment to maximise efficacy, depending on the type of treatment and product	**HBW**	↑	↓	↓/↑	↑
	**170.** Know well the clinical signs and transmission methods of varroosis and viruses	**HBW**	↑	↓	↑	↑
	**171.** Perform at least two treatments per year	**HBW**	↑	↓	↑	↑
	**172.** Rotate the active principles of veterinary medicines to avoid *Varroa* spp. resistance	**HBW**, EW	↑	↓	↑	↑
	**173.** Check the health status of drones producing colonies, especially for viruses	**HBW**	↑	↓	↑	↑
	**174.** Prioritise the use of medicines allowed in organic farming (instead of conventional products) to control *Varroa* spp.	**HBW**, **EW**	=	=	↑	↑
	**175.** Administer varroacide treatments using active ingredients that prioritise bee welfare, such as those with a lower toxicity for honey bees	**HBW**	↑	=	↑	↑
	**176.** Ensure a sufficient number of healthy spare bee colonies are available at the right time, depending on climate and vegetation conditions	**HBW**, EW	↑	↓	↑	↑
	**177.** Try to select and breed colonies that are *Varroa* spp. -tolerant/resistant	**HBW**, HW	↓	↓	↑/↓	↑
	**178.** Properly use formic acid for *Varroa* spp. treatment	**HBW**, **EW**	↑	↓	↑	↑
	**179.** Properly use oxalic acid (including sublimated) for *Varroa* spp. treatment	**HBW**, **EW**	↑	↓	↑	↑
	**180.** Properly use thymol (e.g., applying only when environmental temperature is high enough)	**HBW**, **EW**	↑	↓	↑	↑
	**181.** Properly use other registered medical products with a low environmental impact	**HBW**, **EW**	↑	↓	↑	↑
	**182.** Treat nuclei and swarms (absence of brood) with oxalic or lactic acid	**HBW**	↑	↓	↓/↑	↑
**5. BIOSECURITY MEASURES** American Foulbrood	**183.** Perform the “ropiness test” to confirm a clinical outbreak of AFB in the apiary	**HBW**	NA	↓	NA	↑
	**184.** Use an AFB test (field kit) to confirm a clinical outbreak of AFB in the apiary	**HBW**	NA	↓	NA	↑
	**185.** Quickly manage affected hives	**HBW**	↑	↓	↑/↓	↑
	**186.** Check for *P. larvae* in asymptomatic colonies by laboratory tests (e.g., stored honey in combs, hive debris) to control the disease. Take samples of colonies (hive debris/adult nurse bees/powder sugar/stores of honey in combs) in the winter season to detect *P. larvae* (by PCR method or microbial isolation) to control disease	**HBW**	NA	↓	↓	↑
	**187.** Perform laboratory analysis (isolation and/or PCR) to confirm a clinical outbreak of AFB in the apiary	**HBW**	NA	↓	↓	↑
	**188.** In the case of infected hives, destroy or process wax safely in order to sterilise it	**HBW**	↑	↓	↓	↑
	**189.** Verify the presence of AFB-typical scales (not removable, firmly adherent to the cell wall) to confirm a clinical outbreak of AFB	**HBW**	NA	↓	NA	↑
	**190.** Visually inspect brood combs during work in the colony	**HBW**	=	↓	↑	↑
	**191.** Do not move potentially AFB-infected materials (e.g., frames/bees/feeds) to healthy colonies	**HBW**	↑	=	↑	↑
	**192.** Be mindful of any unusual odour when opening the hive—a foul smell of carpentry glue is commonly associated with suspected cases of the clinical form of AFB	**HBW**	NA	=	↑	↑
	**193.** Disinfect/incinerate all beekeeping equipment (beehives, mating boxes, boards, frames, queen excluders, nucs, combs, wax sterilisation, etc.) of symptomatic hives	**HBW**	NA	↓	NA	↑
	**194.** Disinfect all beekeeping equipment of asymptomatic hives located in AFB outbreaks	**HBW**	NA	↓	NA	↑
	**195.** Increase frequency of hive inspections in asymptomatic colonies (and in other apiaries managed by the same beekeeper), when there is a lab-confirmed positivity for *P. larvae* spores or when clinical signs of the disease are observed in other hives of the same apiary	**HBW**	↑	↓	=	↑
	**196.** In the case of an AFB outbreak, make shook swarms/destroy all symptomatic AFB colonies (minimising their suffering)	**HBW**	↓	↓	↑/↓	↑/↓
	**197.** In the case of an AFB outbreak, make partial shook swarms/destroy all asymptomatic AFB colonies (minimising their suffering)	**HBW**	↓	↓	↓/↑	↓/↑
	**198.** Ensure the prompt notification of American Foulbrood (AFB) cases to adjacent beekeepers	**HBW**, **HW**	↑	↓	↑	↑
	**199.** Establish a group of advisors in the local beekeeping community to help beekeepers manage the spread of diseases	**HBW**	↑	↓	↑	↑
**5. BIOSECURITY MEASURES** European Foulbrood	**200.** Manage affected hives quickly to control disease	**HBW**	↑	↓	↑	↑
	**201.** Search for the presence of removable scales and yellow and contorted larvae to diagnose a suspicious EFB clinical outbreak	**HBW**	NA	↓	NA	↑
	**202.** Perform laboratory analysis (isolation and/or PCR) to confirm the clinical suspicion of EFB	**HBW**	NA	↓	↓	↑
	**203.** Select queen breeders that are free from EFB	**HBW**	↑	↓	NA	↑
	**204.** Disinfect/incinerate contaminated beekeeping equipment (beehives, mating boxes, boards, frames, queen excluders, etc.) from colonies showing symptoms of EFB during a clinical outbreak	**HBW**	↑	↓	↑	↑
	**205.** Increase hive inspections in symptomless colonies when there is lab confirmation of *M. plutonius* or when clinical signs of the disease are observed in other hives of the same apiary	**HBW**	↑	↓	↑	↑
	**206.** Take samples (hive debris/adult nurse bees/powder sugar/stores of honey in combs) from asymptomatic colonies for laboratory testing in winter or in the case of an outbreak to detect the presence of *M. plutonius* (by PCR method or microbial isolation)	**HBW**	NA	↓	↓	↑
	**207.** Use an on-field EFB kit to confirm a clinical outbreak of EFB in symptomatic hives	**HBW**	NA	↓	NA	↑
	**208.** Disinfect/incinerate all beekeeping equipment (beehives, nuc-boxes, mating boxes, boards, frames, queen excluders, etc.) of EFB asymptomatic colonies in the case of a clinical outbreak	**HBW**	↑	↓	↑	↑
	**209.** Be mindful of any unusual odour when opening the hive—a sour smell is commonly associated with suspected cases of in of the clinical form of EFB	**HBW**	NA	=	↑	↑
	**210.** In the case of an EFB outbreak, make shook swarms/destroy all symptomatic EFB colonies (minimising their suffering) to ensure eradication	**HBW**	↓	↓	↑/↓	↑/↓
	**211.** Do not move potentially EFB-infected materials (e.g., frames/bees/feeds) to healthy colonies	**HBW**	↑	=	↑	↑
	**212.** Inform your neighbours about EFB at your apiary	**HBW**, HW	↑	↓	↑	↑
	**213.** In the case of an EFB outbreak, make partial shook swarms/destroy all asymptomatic EFB colonies (minimising their suffering)	**HBW**	↓	↓	↑/↓	↑/↓
**5. BIOSECURITY MEASURES** Nosemosis	**214.** Do not reuse combs (empty or with stores of honey and/or pollen) from depopulated hives (few workers and the queen) or collapsed hives	**HBW**	↑	=	↑	↑
	**215.** Prevent the contamination of artificial water sources with faeces or drowned or dead bees	**HBW**	↑	↓	↑	↑
	**216.** Choose queen breeders from stocks free of *Nosema* spp. contamination	**HBW**	↑	↓	↑	↑
	**217.** Select and breed honey bees resistant to *Nosema* spp. if possible	**HBW**	↑	↓	↑/↓	↑
	**218.** Remove combs with signs of dysentery	**HBW**	↑	↓	NA	↑
	**219.** Collect samples of forager honey bees (or powder sugar or debris) early in autumn or spring to diagnose nosemosis using PCR or microscopic methods	**HBW**	NA	↓	↓	↑
	**220.** Implement appropriate pathogen control (e.g., *Varroa destructor*) to maintain a proper balance between nurse and forager bees in the composition of the bee colony	**HBW**	↑	↓	↓	↑
	**221.** Treat the colony for *Nosema* spp. (if registered/permitted products available in the country) when percentages of infected bees exceed 40%	**HBW**	↑	↓	↑	↑
	**222.** Strengthen and stimulate the colonies in autumn and spring by administering stimulant integrators or feed supplements	**HBW**	↑	↓	↑	↑
**5. BIOSECURITY MEASURES** Aethinosis (if SHB is present in the area)	**223.** Ensure that the bees cover all frames in the hive (no empty space)	**HBW**	↑	↓	↑	↑
	**224.** Do not leave any frames, combs, or other materials outside beehives that could attract *Aethina tumida* (Small Hive Beetle) and serve as a food source.	**HBW**	↑	↓	↑	↑
	**225.** Conduct regular hive inspections periodically to detect and eliminate the parasite (both adults and larvae)	**HBW**	↑	↓	↑	↑
	**226.** Meticulously track hive movements, including hive ID, movement dates, and exact locations	**HBW**, HW	↑	↓	↑	↑
	**227.** Monitor transport conditions by properly isolating beekeeping equipment and preventing the spread of SHB during transport	**HBW**	↑	↓	↑	↑
	**228.** Store combs in a cold chamber at a temperature below 10 °C to prevent the survival of SHB eggs and larval development	**HBW**	↑	↓	NA	↑
	**229.** Provide artificial nutrition in small amounts at a time, allowing bees to consume it quickly (as pollen/protein feed/supplements are a good substrate for SHB reproduction)	**HBW**	↑	↓	↑	↑
	**230.** Only keep healthy, strong colonies in the apiary	**HBW**, HW	↑	↓	↑/↓	↑/↓
	**231.** Trace the movements of supers and wax meticulously	**HBW**, HW	=	↓	↑	↑
	**232.** Use traps to monitor and control SHB presence in the apiary	**HBW**	↑	↓	NA	↑
	**233.** Store combs in a chamber with less than 34% relative humidity to prevent the survival of SHB eggs and larval development	**HBW**	↑	↓	↑	↑
	**234.** Only keep young queens with hygienic behaviour	**HBW**	↑	↓	↑	↑
	**235.** Use a queen bee excluder to prevent the presence of brood in the supers	HW, **HBW**	↑	↓	↓	↑
**5. BIOSECURITY MEASURES** Aethinosis (if SHB is not present in the area)	**236.** Develop a thorough understanding of the morphology of SHB eggs, larvae, and adults	**HBW**, HW	↑	↓	NA	↑
	**237.** Develop a thorough understanding of hive inspection methods to detect SHB	**HBW**, HW	↑	↓	NA	↑
	**238.** Do not leave frames, combs, or other material outside beehives that may attract and serve as a food source for *A. tumida*	**HBW**	↑	↓	↑	↑
	**239.** Keep only healthy and strong colonies in the apiary	**HBW**, HW	↑	↓	↑/↓	↑/↓
	**240.** Keep only young queens with hygienic behaviour	**HBW**	↑	↓	↑	↑
	**241.** Do not transport live material at risk (such as hives, queens, nucs, etc.) from areas where SHB is present into the apiary	**HBW**, EW	↑	=	↑	↑
	**242.** Do not transport at-risk materials (supers, wax, pollen, etc.) from areas where SHB is present into the apiary	**HBW**, EW	↑	=	↑	↑
	**243.** Ensure that the bees cover all frames in the hive (no empty space)	**HBW**	↑	↓	↑	↑
	**244.** Adopt specific traps for the rapid visual detection of SHB	**HBW**	↑	↓	NA	↑
	**245.** Periodically monitor the presence of SHB by sampling debris or honey	**HBW**	↑	↓	NA	↑
	**246.** Use a queen bee excluder in order to avoid the presence of brood in the supers	HW, **HBW**	↑	↓	↓	↑
**6. ANIMAL** **FEEDING AND WATERING**	**247.** Do not feed the bees with honey, pollen, or supplements, unless the absence of pathogens (such as spores of AFB, chalkbrood, Nosema, EFB, etc.) has been certified	**HBW**	↑	↓	↑	↑
	**248.** Provide artificial feeding, supplements, and water during periods of shortage or when necessary, but avoid their use when not necessary	**HBW**	↑	↓	↑	↑
	**249.** Wintering: ensure that an adequate amount of stores is present in the hive	**HBW**	↑	↓	↑	↑
	**250.** Ensure that an adequate food supply is provided to nuclei and swarms, when needed	**HBW**	↑	↓	↑	↑
	**251.** Ensure that the bees have access to safe water sources	**HBW**	↑	↓	↑	↑
	**252.** Avoid feeding bees openly in the field to prevent robbing and the spread of diseases	**HBW**	↑	=	↑	↑
	**253.** If robbing occurs, temporarily close the hive or reduce the entrances, clean honey residues from hives surfaces or surrounding areas, and use the smoker	**HBW**	↑	↓	↑	↑
	**254.** Use only feed safe for bees (e.g., specifically registered, without bee pathogens, etc.)	**HBW**	↑	=	↑	↑
	**255.** Clean the feeders after their use	**HBW**	↑	↓	↑	↑
	**256.** Do not heat sugar solutions over tap-warm temperature	**HBW**	↑	=	↑	↑
	**257.** Provide a constant water source	**HBW**	↑	↓	↑	↑
	**258.** Use a proper feeder	**HBW**, HW	=	=	↑	↑
	**259.** Provide adequate watering during transport if necessary	**HBW**	↑	↓	↑	↑
**7. RECORD** **KEEPING**	**260.** Keep records of veterinary medicinal treatments	**HBW**	↑	↓	↑	↑
	**261.** Beekeeper and hives should be recorded in the National Beekeeping Registry	**HBW**, HW	=	NA	↑	↑
	**262.** Record the exact position of the bee yards	**HBW**, HW	=	↓	↑	↑
	**263.** Identify with the numbers/letters all the hives in each apiary	**HBW**	=	↓	↑	↑
	**264.** Keep records of honey bee diseases and colony mortality or depopulation	**HBW**, HW	↑	↓	↑	↑
	**265.** Set up a data-recording system that can be used to trace exactly which batches of commercial feed the colonies were fed with	**HBW**, HW	=	↓	↑	↑
	**266.** Retain all documents/certificates related to the commercial feed used	**HBW**	=	↓	↑	↑
	**267.** For each colony or group of colonies, obtain and retain all commercial and health documents, ensuring their exact traceability from the farm or establishment of origin to their final destination	**HBW**	↑	↓	↑	↑
	**268.** Record the movements of all reared colonies, ensuring that incoming colonies are traceable to their source	**HBW**	=	↓	↑	↑
	**269.** Keep records of the movements of hives, swarms, and queen bees	**HBW**	=	↓	↑	↑
	**270.** Record the period of hive product collection from each apiary	**HBW**	↑	↓	=	=
	**271.** Keep detailed records of the origin and use of all medicines, including batch numbers, dates of administration, doses, treated hives, and withdrawal times; treated hives or apiaries should be clearly identified	**HBW**	↑	↓	↑	↑
	**272.** Retain all documents/certificates that indicate the raw materials used in feed manufactured by the beekeeper and given to the colonies	**HBW**	=	↓	↑	↑
	**273.** Create a unique identification number/code for the apiary and beehives’ identification to easily trace/refer to them	**HBW**, **HW**	=	↓	↑	↑
	**274.** Keep records of breeding activities (e.g., all breeding stock, queens’ birth dates, their origin and arrival, the breeding dates, and outcomes in cases of instrumental insemination, etc.)	**HBW**, **HW**	=	↓	↑	↑
	**275.** Establish a data-recording system to ascertain the exact origin (batch) of bee products produced	**HW**	=	↓	=	=
	**276.** Keep all documents related to self-checks and official controls regarding the proper management of colonies and the sanitary and hygienic quality of bee products	HBW, **HW**	↑	↓	↑	↑
	**277.** Record datasheets for each detergent/disinfectant used	**HBW**, **HW**, **EW**	=	↓	=	=
	**278.** Record the disinfection procedures used	**HBW**, **HW**, **EW**	=	↓	=	=
	**279.** Retain any document confirming the analytical aspects of any feed or water administered to bees	**HBW**	↑	↓	↑	↑
	**280.** Keep records of the origin and batches of all feed used in the apiary, as well as their leaflets	**HBW, HW**	=	↓	↑	↑
	**281.** Keep a list of certified suppliers	**HW**	=	↓	=	↑
	**282.** Record any other management changes that may occur	**HBW**	↑	↓	↑	↑
	**283.** Record any change in feeding	**HBW**	↑	↓	↑	↑
	**284.** Keep records of harvesting data	**HW**	↑	↓	=	=
	**285.** Use a recording method (physical or digital registers/apps) that fits to your needs and the size of your beekeeping operation	**HW**, **HBW**	↑	↓	↑	↑
	**286.** During hive inspections, record the characteristics of honey bee queens (e.g., brood pattern, amount of eggs laid, cohesiveness of the colony, etc.)	**HBW**, **HW**	↑	↓	=	↑
	**287.** During hive inspections, record any zootechnical performance of the colony (productivity, hygienic behaviour, docility, etc.)	**HBW**, **HW**	↑	↓	=	↑
	**288.** Mark/identify hives that need subsequent attention	**HBW**, **HW**	=	↓	↑	↑
	**289.** Keep record of the veterinary medicinal products authorised in your country	**HBW**, **HW**	↑	↓	↑	↑
	**290.** Periodically update the list of the authorised veterinary medicinal products	**HBW**, **HW**	↑	↓	↑	↑
	**291.** Keep all laboratory reports, including bacteriological tests and sensitivity	**HBW**, HW	=	↓	↑	↑
**8. TRAINING**	**292.** Attend a personal training programme in beekeeping	**HBW**, **HW**	↑	↓	↑	↑
	**293.** Conduct training/improve knowledge on honey bee diseases and clinical signs	**HBW**, **HW**	↑	↓	↑	↑
	**294.** Retain documents certifying the qualification and training of individuals working with bees	**HBW**, **HW**	=	↓	=	=
	**295.** Attend personal training on One Welfare, with a focus on honey bee welfare	**HBW**, **HW**, **EW**	↑	↓	↑	↑
